# Spatiotemporal Heterogeneity of Alzheimer's Disease‐Related Functional Brain Network Dedifferentiation

**DOI:** 10.1002/alz70856_107430

**Published:** 2026-01-11

**Authors:** Roy Massett, Ziwei Zhang, Micaela Chan, Gagan S Wig, Alzheimer's Disease Neuroimaging Initiative (ADNI), Wig Neuroimaging Lab

**Affiliations:** ^1^ University of Texas at Dallas, Richardson, TX, USA; ^2^ University of Texas Southwestern Medical Center, Dallas, TX, USA; ^3^ Laboratory of Neuro Imaging (LONI), University of Southern California, Los Angeles, CA, USA

## Abstract

**Background:**

Alzheimer's disease (AD) is a highly heterogeneous condition both in terms of the distribution of pathology deposition and clinical manifestation. The organization of functional brain networks is related to AD pathology, with recent work showing that higher dementia severity is related to the desegregation of brain networks (Zhang *et al*., 2023). However, the spatiotemporal heterogeneity of these changes in network segregation and how they contribute to different cognitive deficit profiles remains an open area of research. To contribute to this question, we applied a clustering‐based data‐driven disease progression model to system‐level measures of network organization.

**Method:**

We included 754 resting‐state functional magnetic resonance imaging (fMRI) scans from cognitively impaired individuals (CDR > 0) enrolled in the Alzheimer's Disease Neuroimaging Initiative. The correlations of fMRI resting‐state time series between nodes were used to construct brain networks (Chan *et al*., 2014). Nodes were assigned to a functional system based on a previously defined functional atlas (Power *et al*., 2011). Network segregation was calculated for each brain system as measures of system‐level organization. The SuStaIn algorithm was used to simultaneously stage and subtype subjects according to their patterns of network segregation (Young *et al*., 2018). Functional systems that were clustered together were further analyzed collectively using linear regression models.

**Result:**

SuStaIn identified two subtypes of alterations in system‐level network segregation, which were upheld in *k*‐fold cross validation. One cluster showed decreases in the segregation of sensory‐motor systems and several additional systems, including the superior temporal gyrus, salience, and medial temporal parietal systems. The other cluster showed decreases primarily in segregation of association systems including the default mode network, task control systems, attention systems, and memory retrieval systems. Further, we observed a significant interaction effect between system‐type and model‐estimated disease stage and subtype.

**Conclusion:**

Resting‐state fMRI signals can be used to identify dissociable patterns of AD‐related brain network desegregation relating to different profiles of brain network dysfunction and cognitive impairment. These findings begin to describe the spatiotemporal heterogeneity in brain network decline, and their relation to cognitive trajectories, which is relevant not only to AD prognostics but also evaluating clinical trial outcomes in cognitively‐diverse patient populations.

